# Risk of Vulvar HSIL and Penile Neoplasias in Anogenital Lichen Planus: A Case-Control Study

**DOI:** 10.1097/LGT.0000000000000879

**Published:** 2025-02-14

**Authors:** Niina K. Hieta, Lotta Tapana, Jenni M. Söderlund, Jutta K. Huvila, Lauri A.I. Talve, Marjut A.M. Haataja

**Affiliations:** 1 Department of Dermatology; 2 Auria Clinical Informatics; 3 Department of Obstetrics and Gynaecology; 4 Department of Pathology, University of Turku and Turku University Hospital, Turku, Finland

**Keywords:** lichen planus, human papillomavirus infection, squamous cell carcinoma, SCC, VIN, dVIN, PeIN, HSIL

## Abstract

**Objective:**

Lichen planus (LP) is a chronic inflammatory skin disease that may affect the skin, scalp, nails, and mucosa. The aim of this study was to investigate the risk of genital premalignant and malignant conditions in patients with anogenital LP (agLP).

**Methods:**

The authors identified 60 male and 56 female patients with agLP by searching the biobank archives for a genital or perianal skin biopsy showing LP between 2004 and 2020. They also included 10 randomly selected age- and sex-matched controls for each patient. The risks of genital cancers and their precursors were evaluated.

**Results:**

The incidence of agLP was 1.54 per 100,000 men and 1.36 per 100,000 women. There was no statistical difference between male and female incidence (*p* = .5721). The odds ratio (OR) for high-grade squamous intraepithelial lesion (HSIL) of the vulva was 31.2 (95% CI = 2.46–1645.00; *p* = .01). The OR for penile neoplasias could not be calculated because control patients had no neoplasias, but the difference was suggestive of statistical significance (95% CI = 1.90–infinite; *p* = .008). The mean age at the time of diagnosis of agLP was 59.9 years (median 62 years) in female patients and 40.8 years (median 33 years) in male patients. The difference in the mean ages of female and male patients was statistically significant (95% CI = 11.92–26.13; *p* < .001).

**Conclusions:**

Patients with agLP may have an increased risk for vulvar HSIL and penile neoplasia.

Lichen planus (LP) is a chronic inflammatory skin disease affecting the skin, scalp, nails, and mucosa.^1^ The incidence of LP has been estimated to be between 0.14% and 1.27% of the general population, and at least two thirds of cases are thought to occur between the ages of 30 and 60 years.^1^ Few studies have investigated the age of onset or incidence of anogenital LP (agLP).^2^

The majority of vulvar cancers (VCs) develop either from human papillomavirus (HPV) infection or as a result of a chronic skin disease.^3^ Vulvar lichen sclerosus (VLS) has been shown to be a major risk factor of VC. The risks of VC and high-grade squamous intraepithelial lesion (HSIL) in patients with VLS are 2.2% and 0.4%, respectively, with the risk of VC being higher in patients who do not comply with treatment.^4,5^ From a systematic review by Vieira-Baptista et al., the risks of VC and HSIL in patients with vulvar lichen planus (VLP) are 3.0% and 1.4%, respectively, when taking into consideration only the patients with VLP and not all types of LP.^4^ VLP may also increase the risk of recurrence of vulvar HSIL.^6^ It is not known whether VC in patients with VLP develops through differentiated vulvar intraepithelial neoplasia (dVIN), as in lichen sclerosus (LS), or through a HPV-associated pathway via an HSIL. Vulvar lichen sclerosus also seems to be a risk factor for vulvar melanoma,^7^ but there are no reports of vulvar melanoma in patients with agLP.

Penile precursor lesions and tumors are classified into HPV-associated and HPV-independent types.^6–9^ Human papillomavirus and LS are the most important risk factors of penile intraepithelial neoplasm (PeIN).^10^ While it has been shown that penile lichen sclerosus is a known risk factor for penile carcinoma,^11^ the role of penile LP has been less studied. In one study, biopsy-proven LP was found in 26% (9 out of 35) of HPV-negative penile squamous cell carcinomas (SCCs).^12^ Apart from these, only some case reports have been published on the connection between LP and penile SCC.^13–16^ In a study of 35 patients with PeIN, LP was found in 2 patients with bowenoid papulosis and 1 patient with erythroplasia of Queyrat.^17^ A population-based registry study found the odds ratio (OR) of PeIN in patients with any type of LP to be 12.0.^18^

In this study, we investigated the risk of neoplasias in female and male patients with agLP and the age at the time of diagnosis of agLP in these patients. The study was performed in a well-being services county consisting of about 490,000 inhabitants. The university hospital of the county and its pathology department also serve as a tertiary hospital for 2 other counties. Data on the ethnicity of the patients is not collected in the electronic health records, but the population is mainly White, non-Hispanic, and the male population is mainly uncircumcised.

## METHODS

Patients with an agLP diagnosis were identified by searching for histological samples of agLP during the period from January 1, 2004, to December 31, 2020, in the biobank archives. The histopathological diagnosis had to be LP. Topographical sites given as penis, foreskin of penis, glans penis, perineum, vulva, clitoris, external genitalia, labium majus, labium minus, vagina, portio, perianal skin, and anus were included.

Two pathologists (one dermatopathologist and one gynecologic cancer specialist) reevaluated the histological samples showing agLP in patients with diagnoses for coincidental LS and agLP, and also the samples showing agLP in patients with genital neoplasias in cases where the diagnosis of agLP was uncertain. For review of the samples, study approval was obtained from the biobank, the ethics committee, and the clinical research center.

The yearly incidence rates were calculated based on the yearly number of diagnoses of agLP separately in female and male patients and the yearly number of female and male inhabitants in our hospital catchment area. The general incidence rate for female and male patients was calculated as the total number of diagnoses divided by the total sum of yearly numbers of female and male inhabitants in our hospital catchment area.

We also included a randomly selected control group from the hospital's electronic health records of 10 age- and sex-matched controls for each identified patient. The control patients were patients who had been treated in the hospital for any reason and who had no diagnosis of LP.

### Statistical Analyses

Continuous variables are described using mean and SD if normally distributed, or median and lower and upper quartiles (Q1–Q3) if non-normally distributed. Discrete variables are presented using observed frequencies and proportions. We analyzed the association of LP with other diseases using Fisher exact test. The results are presented as ORs with 95% CIs and *p* values. Cross-tabulation was used to examine the difference in incidence between the sexes, and the significance of the difference between the 2 proportions was assessed by Pearson chi-square test with Yates continuity correction. When comparing the patients' age at first diagnosis, the equality of variances of the groups was checked with the F test. The variances were different, so group means were compared using Welch *t*-test. The difference in the median age of diagnosis was analyzed with the Wilcoxon rank sum test with continuity correction. Statistical analyses were performed, and tables, figures, and listings were produced using R version 4.3.3 (R Core Team, 2018) with RStudio Server in Auria Clinical Informatics secure operating environment.

## RESULTS

In our first dataset, we found 66 female and 61 male patients with a diagnosis of LP confirmed by genital or perianal histological biopsy. Among those patients, there were 7 patients with vulvar carcinomas (10.6% of female patients), 6 patients with vulvar HSIL (9.1%), 2 patients with penile carcinomas (3.3% of male patients), and 1 patient with penile PeIN (1.6%). Based on reevaluation of histology, written patient records, and clinical photos, we excluded 11 patients from the study. Six women with agLP originally also had histologically confirmed VLS. However, after reevaluation, all 6 turned out to have only LS and were excluded from the study. Therefore, there were no patients who actually had both LP and LS. In addition, 1 female and 1 male patient with a previous diagnosis of LP alone turned out to have LS instead, and 3 female patients had unspecific inflammatory changes misleading for LP. All of the vulvar SCCs, 3 vulvar HSILs, and 1 penile SCC were among the excluded cases.

**FIGURE 1:**
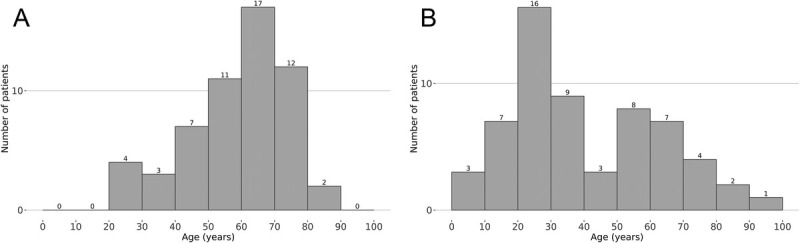
Age at the time of diagnosis of anogenital lichen planus in (A) female and (B) male patients.

We identified a total of 56 female and 60 male patients with agLP. The incidence of agLP was 1.54 per 100,000 men and 1.36 per 100,000 women. There was no statistical difference between male and female incidence (*p* = .5721). The mean age at the time of diagnosis of agLP was 59.9 years (median = 62 years, range = 24–88 years) for female patients and 40.8 years (median = 33 years, range = 8–91 years) for male patients (Figure 1). The differences in the mean and median ages between female and male patients were statistically significant (for mean age, 95% CI = 11.92–26.13, *p* < .001; and for median age, 95% CI = 11.00–36.00, *p* < .001). The most common age of diagnosis was 60–69 years for female patients and 20–29 years for male patients. Ten male patients were under the age of 20, whereas the youngest female patients were 24 years old.

Among the 56 female patients with agLP, 3 patients (5.3%) (patients 1–3) had HSIL (Table 1). The OR was 31.2 (95% CI = 2.46–1645.00, *p* = .01) for HSIL. None of the female patients had dVIN or SCC. Of our 3 female patients, 2 (patients 1 and 2) also had vulvar condyloma confirmed with a biopsy.

**TABLE 1:** Female and Male Patients With Lichen Planus and Squamous Cell Carcinoma (SCC), High-Grade Intraepithelial Neoplasia (HSIL), or Penile Intraepithelial Neoplasia (PeIN)

Patient no. and sex	Age at dg of dysplasia	Age at dg of LP	Time from dg of dysplasia to dg of LP (y)	Type of dysplasia	Treatment of HSIL/SCC	Treatment of LP	Other diseases	Smoking	Condyloma/hrHPV test	No. treatments (surgery/PDT)
1/F	53	53	0	HSIL (in situ carcinoma, VIN3)	Surgery	No (affected area removed in surgery)	Asthma	Yes	+/ND	1/0
2/F	68	60	−8	HSIL (VIN3)	Surgery	Clobetasol ointment	No?	No	+/−	1/0
3/F	79	69	−10	HSIL (VIN2)	Surgery	Clobetasol, betamethasone, and tacrolimus ointments	Asthma, rectal cancer, oral LP	No	−/−	1/0
4/M	74	76	2	SCC, PeIN	Circ and local excisions	Acitretin, clobetasol	–	ND	+/ND	5/0
5/M	57	58	1	PeIN (carcinoma in situ, bowenoid papulosis –type)	Circ and PDT	Acitretin, clobetasol	HDT, gastritis, gonarthrosis, pollen allergy	ND	+/ND	1/3

Circ indicates circumcision; Dg, diagnosis; F, female; hrHPV, high-risk human papilloma virus; HTD, hypertensive disease; M, male; ND, not done; PDT, photodynamic therapy, always performed twice; VIN, vulvar intraepithelial neoplasia.

**FIGURE 2:**
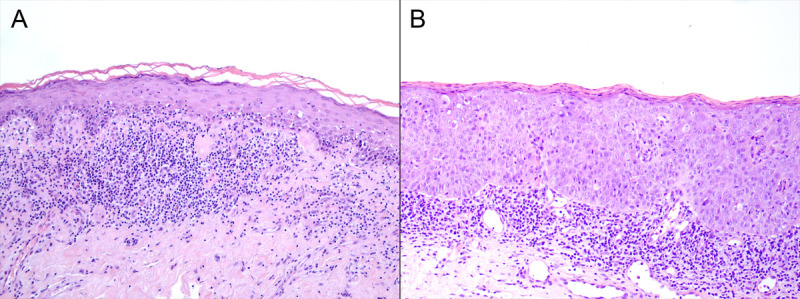
Photomicrographs (H&E × 100) of biopsies from patient 4. A, Lichenoid inflammation with apoptotic bodies at rete ridges and vacuolar change consistent with lichen planus. B, Parakeratotic hyperkeratosis, loss of maturation, nuclear hyperchromasia, and mitotic activity in the upper layers of the epidermis consistent with high-grade squamous intraepithelial lesion.

Among the 60 male patients with agLP, 2 patients (patients 4 and 5) had penile neoplasias. Patient 4 had been treated twice for SCC (first in the foreskin and then in the frenulum) in private practice before being remitted to hospital. He had 3 HPV-associated PeINs, occurring twice in the glans and then in the frenulum, during the first years of follow-up in the Department of Urology and later upon admission to the Department of Dermatology (Figure 2). The histology of the first neoplastic change in patient 5 was PeIN, suggestive of HPV-related bowenoid papulosis despite the unusually older age of the patient for this type of dysplasia. He also later had PeIN in the glans inside an area of condyloma, and later HPV-associated PeIN in the sulcus area. The diagnosis of LP was done at the same time with or after the diagnosis of dysplasia in both patients (Figure 2). Neither of the patients had concomitant LS. Neither high-risk HPV (hrHPV) nor p16/p53 status was investigated, but both patients had a histological diagnosis of condyloma during follow-up. The electronic health records did not have information on the patients' smoking status. In both patients, the disease activity abated after dermatological treatment was started.

The ORs for the risk of SCC or PeIN in male patients with agLP could not be calculated because there were no patients with penile neoplasias in the control group, which was created in the same way as for female patients. However, when these 2 patients were pooled together, the result was statistically significant for keratinocyte-type premalignancies or malignancies (*p* = .02).

## DISCUSSION

Of 56 female patients with agLP, none had VC, but 3 (5.4%) had HSIL when reclassified according to the current classification of vulvar squamous intraepithelial lesions (OR = 31.2).^19^ The risk for VC had been higher in previous studies, where 0.9%–2.3% of patients with vulvar LP developed some type of VC, as reviewed by Vieiro-Baptista et al.^4^ Vulvar HSIL in our patients was more common than the combined rate of 1.4% from previous studies.^4^ The number of diagnoses of HSIL with no dVIN suggests that in LP, the carcinogenesis may occur through a different pathway than in LS, where dVIN is the precursor of vulvar squamous cell carcinoma.^4^

Among the 60 male patients with agLP, 2 had PeIN, and 1 of them also penile SCC. Because none of the control patients had penile neoplasias, the OR for PeIN or penile SCC could not be calculated, but there was a statistically significantly increased risk for keratinocyte-type neoplasias. Our results are in line with those of a previous study where the OR of PeIN in patients with any type of LP was 12.0.^18^ There have not been studies on the risk of penile SCC in patients with penile LP.

Reevaluation of our original dataset showed that all 6 women with a histological diagnosis of both agLP and VLS only had VLS alone, and were excluded from the study. There are few publications on patients with both VLS and VLP.^20^ Some variants, especially the inflammatory variant of LS, may mimic LP.^21^ This may explain the previous histopathological misdiagnoses in our study and emphasizes the importance of the involvement of an experienced vulvar pathologist.

All except 1 of our 5 patients with LP and neoplasia (2 female and both male patients) had also a biopsy-proven condyloma, usually diagnosed after the diagnosis of neoplasia. The male patient with 3 PeINs (patient 5) first had a histological diagnosis of in situ carcinoma, suggestive of bowenoid papulosis, and later PeIN inside a large condyloma. Condylomas are usually caused by low-risk HPV and do not contribute to cancer risk. However, in penile cancer, low-risk viruses usually causing condylomas are more common, as reviewed by Silva et al.^22^ There are no such connections in vulvar, vaginal, or oral cancers.^22^ On the other hand, oncogenic HPV genotypes including HPV 16 and 18 have been found in condylomas.^23^ It is possible and not even rare to have different types of HPV simultaneously. These results suggest that LP and even a low-risk HPV together may increase the risk of malignant transformation.

It is not known how LP and HPV might be connected. It has been suggested that epithelial ulcers make HPV infection more likely. In support of this, a meta-analysis has shown that the risk of oral HPV infection is increased in patients with oral LP, especially in those with erosive lesions.^24,25^ However, results related to the possible association between HPV and LP in malignant transformation have been conflicting and mainly focused on hrHPV types.^25^ Human papillomavirus has also been shown to be common in patients with LS,^26,27^ even though LS is usually not an erosive disease. In a study of 345 male patients with PeIN, clinical or histological evidence of both HPV and lichen sclerosus was present in 29.4% of cases.^10^ In a study of 89 male patients with genital LP, bowenoid HPV-dependent intraepithelial neoplasia was absent at diagnosis but developed during follow-up in 2 patients.^28^ The treatment of LP with immunosuppressive medications such as corticosteroids leading to upregulation of HPV replication might explain the connection between LP and HPV. It is possible that in patients 2–4 the development of condylomas was provoked by topical corticosteroid treatment and had no connection with the HSIL or cancer, as they appeared only after the diagnosis of neoplasia in these patients. Patient 5 had a clinical suspicion of condyloma and a biopsy-proven bowenoid papulosis before later treatments with corticosteroid creams. In patients with VLS, treatment with high-potency topical corticosteroids does not seem to increase the risk of HSIL recurrence after the first HSIL.^29^

Many of the previous studies on the risk of vulvar premalignancies or malignancies in patients with LP have been done in patients who were diagnosed and treated for LP in specialist clinics, and thus informed about the risk of malignancies in patients with active, ongoing treatment. A previous study pointed out that all the women with VLP who developed dVIN or VC had severe disease and responded poorly to treatment.^30^ In our study, 1 out of 3 female patients (33%) had not been diagnosed with LP prior to the diagnosis of HSIL. Two patients (67%) had received a diagnosis of LP but were not followed up in a specialized vulvar clinic, and the treatment with ultrapotent corticosteroids was occasional and symptomatic rather than active maintenance therapy. In fact, none of the patients that had been followed in specialized vulvar appointments in dermatology or gynecology clinics in our hospital were among the patients with a diagnosis of HSIL. Neither of the 2 male patients in this study were diagnosed with LP before the diagnosis of penile neoplasia. The patient with penile SCC had no further dysplastic changes after beginning active treatment for LP. The patient with penile PeINs had 1 occurrence of PeIN shortly after beginning treatment for LP and another PeIN 2 years later, followed by 6 years of stable condition. This suggests that patients with genital symptoms should receive the correct diagnosis of genital dermatosis and active treatment aimed at preventing the inflammatory activity to avoid malignant transformation.

The median age at the time of histopathological diagnosis of agLP in female patients was 62 years (range = 24–88 years), which is 11 years more than in a previous study on vulvar erosive LP.^31^ The difference may be due to our patient group including all types of agLP, not just the erosive type. The mean age was 59.9 years, and the most common age of diagnosis was 60–69 years. The age at the time of diagnosis of agLP in female patients in our patient group was comparable to that in a previous report studying LP at all anatomical sites and to that of patients with LS.^27,32^

In male patients, agLP seemed to occur at a younger age than usually thought, the mean age being 41 years and the range 8–91 years. In a previous study of 89 male patients with genital LP, the mean age was 51 years,^28^ 10 years more than in our patients. The age distribution in our patient group peaked at 20–29 years. There was also another smaller peak of patients 50–69 years of age. The difference in the mean age of female and male patients at the time of diagnosis was statistically significant.

The high incidence of agLP in boys and young adult males is similar to that shown for LS. In contrast, there were no female pediatric patients, the youngest female patient being 24 years of age. In a previous study by Mohandesi et al. of LP in 26 patients under the age of 20 years, there were 4 cases of genital-only LP, all in boys, with a mean age of onset of 18.3 years.^33^ Another study with 28 vulvar LP patients found 2 pediatric patients aged 13 and 17 years.^21^ Our results are in accord with the study of Mohandesi et al., with no cases of genital LP in girls under 24 years of age. This suggests that the etiologies of agLP and LS are at least partly different.

The incidence of agLP was 1.54 per 100,000 men and 1.36 per 100,000 women. There was no statistical difference between male and female incidence (*p* = .5721). This is in contrast to LS, where the incidence among female patients is higher than among male patients. To our knowledge, this the first study directly comparing the incidence of agLP between females and males.

Limitations of this study include its retrospective nature and the fact that it included only patients who were remitted to University Hospital and who also had a histological confirmation of agLP. Thus, patients with less severe symptoms who were treated in primary health care or by private practice specialists were not in the study population. AgLP can be clinically diagnosed in clear cases, and a definite histopathological diagnosis is not always possible to obtain from a biopsy sample.^28^ Therefore, it is likely that not all patients treated for agLP in our hospital were included in this study. However, all of the patients in this study did have a histologically confirmed diagnosis of agLP.

## CONCLUSION

In our study, the number of patients was relatively small, which limits the generalizability of the results. However, based on our findings, agLP appears to be a risk factor for developing genital neoplasias in both female and male patients. It is not known whether active treatment can reduce the risk of malignant transformation in agLP. However, in our patients, the neoplasias developed before the start of active disease-modifying treatment. In LS, a disease with many similarities to agLP, active treatment aiming to reduce disease activity and restore normal skin structure has been shown to reduce the risk of malignant transformation; our results suggest that this may also be warranted in patients with agLP. The possibility of agLP should be taken into consideration among boys and young adult male patients with balanitis of unclear origin.
